# An Improved Sliding Window Area Method for *T* Wave Detection

**DOI:** 10.1155/2019/3130527

**Published:** 2019-04-01

**Authors:** Haixia Shang, Shoushui Wei, Feifei Liu, Dingwen Wei, Lei Chen, Chengyu Liu

**Affiliations:** ^1^School of Control Science and Engineering, Shandong University, Jinan 250061, China; ^2^School of Instrument Science and Engineering, Southeast University, Nanjing 210096, China; ^3^Department of Electronic & Electrical Engineering, Bath University, Bath BA27AY, UK; ^4^School of Science and Technology, Shandong University of Traditional Chinese Medicine, Jinan 250355, China

## Abstract

**Background:**

The *T* wave represents ECG repolarization, whose detection is required during myocardial ischemia, and the first significant change in the ECG signal is being observed in the ST segment followed by changes in other waves like *P* wave and QRS complex. To offer guidance in clinical diagnosis, decision-making, and daily mobile ECG monitoring, the *T* wave needs to be detected firstly. Recently, the sliding area-based method has received an increasing amount of attention due to its robustness and low computational burden. However, the parameter setting of the search window's boundaries in this method is not adaptive. Therefore, in this study, we proposed an improved sliding window area method with more adaptive parameter setting for *T* wave detection.

**Methods:**

Firstly, *k*-means clustering was used in the annotated MIT QT database to generate three piecewise functions for delineating the relationship between the RR interval and the interval from the *R* peak to the *T* wave onset and that between the RR interval and the interval from the *R* peak to the *T* wave offset. Then, the grid search technique combined with 5-fold cross validation was used to select the suitable parameters' combination for the sliding window area method.

**Results:**

With respect to onset detection in the QT database, *F*1 improved from 54.70% to 70.46% and 54.05% to 72.94% for the first and second electrocardiogram (ECG) channels, respectively. For offset detection, *F*1 also improved in both channels as it did in the European ST-T database.

**Conclusions:**

*F*1 results from the improved algorithm version were higher than those from the traditional method, indicating a potentially useful application for the proposed method in ECG monitoring.

## 1. Introduction

Nowadays, an increase in the number of people suffering from heart diseases has been seen. Characterized by several waveforms such as the *P* wave, QRS complex, and *T* wave, electrocardiogram (ECG) becomes the most intuitive and basic tool to diagnose heart diseases in clinical applications which can provide essential physiological/pathological information for clinical diagnoses and decision-making [1], including important time interval information between the onset and offset of different waves [2]. Besides, many wearable monitoring devices have appeared in recent years, which makes it possible to monitor ECG signals throughout an individual's daily life. Meanwhile, a large amount of ECG data are generated daily, which is impossible for physicians to view/diagnose each ECG signal manually [3]. Therefore, developing accurate automatic analysis algorithms for ECG signals is critical, especially with respect to mobile ECG monitoring [4]. Furthermore, QRS complex have been widely investigated because of its highest amplitude over the past decades. Up to now, there are many classical methods for detecting QRS complex and most of the methods have been listed in [5], and the classical widely-used methods are parabolic fitting [6], neural-network-based method [7], and convolutional neural network [8]. In addition, those methods for detecting the QRS complex have shown high sensitivity with positive predictivity (>99%) on the MIT-BIH arrhythmia database [9], which can provide powerful support for other waves' detections.

As one of three main waves of ECG, the *T* wave represents ECG repolarization, and its absence or unusual shapes may signify disruption in repolarization or another segment of the heartbeat [10]. Additionally, *T* wave abnormalities are associated with some heart diseases such as inverted *T* waves found in other leads (other than the V1 to V4 leads), which is related to an increase in cardiac deaths, and a tall or wide QRS complex with an upright *T* wave is further suggestive of a posterior infarction. Furthermore, during myocardial ischemia, the first significant change in ECG signal is being observed in ST-segment followed by changes in other waves like *P* wave and QRS complex of ECG signal. Hence, detection of the *T* wave is significant in clinical applications [11].

However, accurate/robust *T* wave detection still presents challenges due to its low amplitude (usually 0.1 to 0.3 mV) as well as great variations in *T* waves' morphologies [12], like positive *T* wave, negative *T* wave, and biphasic *T* wave. Besides, most of the ischemic cases suffering from earlier STEMI (ST-elevation myocardial infarction) have a prominent ST elimination or depression, which significantly affects the detection of the *T* onsets. Nowadays, various approaches based on different techniques have been proposed for *T* wave detection, and those typical techniques are wavelet [13, 14], mathematical model [15], support vector machine (SVM) [16], artificial neural network (ANN) [17–19], low-pass differentiation (LPD) [20], hidden Markov model (HMM) [21, 22], partially collapsed Gibbs sample and Bayesian (PCGS) [23], “wings” function [24], derivative curve [25], adaptive technique [26], computing the Trapezium's area [27], TU complex analyses [28], correlation analysis [29], *k*-nearest neighbor [30], and sliding window area (SWA) [31]. In these aforementioned methods, the wavelet-based method is robust to waveform morphological variations but is sensitive to noise [13, 14]. The mathematical model method needs to build robust ECG templates, but when the waveform variations are large, building universal templates becomes difficult [15]. The SVM-based method is efficient but constructing efficient features is tough [16], and the ANN-based method faces the drawback of high computational complexity [17]. As a comparison, the SWA method has low computational complexity which is also robust to noise and waveform morphological variations [31].

In 2006, Zhang et al. first proposed the SWA method for detecting *T* wave offsets and confirmed its efficiency in the QT database [31]. Subsequently, Song et al. improved this method for detecting *T* wave onsets [32]. Afterwards, our team combined onsets and offsets detection for classifying the morphology of the ST segment [33]. In 2017, our team analyzed its efficiency in the QT database with a different evaluation index (*F*1 measure), and we found that there is still some space for further improvement since the parameter settings in the transitional SWA method are not adaptive [34], and the parameters given by Zhang et al. [31]and Song et al. [32] are empiric values and there is no optimization step included.

Hence, in this study, an improved SWA method for both onset and offset detections of *T* wave with more adaptive parameter settings is proposed. The performance of the improved method was compared with the traditional method, and both methods were validated in two common ECG databases: (1) the QT database (training and testing) and (2) another independent European ST-T database (only testing).

## 2. Methods

### 2.1. Data

Records from two datasets are used. The first is the QT database, which contains 105 15-minute two-channel ECG recordings with the sample rate of 250 Hz, and we chose it as the training and testing sets because multiple-type records from different databases are contained in this database. Besides, totally 43 recordings have manually annotated *T* wave onsets and 103 recordings have manually annotated *T* wave offsets. All records with annotations are selected, and for each record, a 0.05–45 Hz low-pass zero-phase filter was applied for denoising before importing to our algorithm. Furthermore, there are usually 30 to 100 representatively manually annotated discrete beats in each annotated recording. Thus, an RR interval adjustment is also needed before using these records because we used the manually annotated *R* peak locations.  Table 1 shows the summarized annotated information of the QT database. More detailed information about the annotations of this database can be found in the study by Laguna et al. [35].

The second database is the European ST-T database, which consists 90 2-hour two-channel ECG recordings sampled at 250 Hz, and records of this database are only used to test the robustness of our improved method. The European ST-T database is chosen because of its widely usages in evaluation of algorithms for analysis of ST and *T* wave changes [36, 37]. In this study, 23 recordings (only the first 5 minutes in each recording) were selected and were manually annotated for *T* wave onsets and offsets by a trained staff member because of loss of *T* wave international annotations.  Table 1 also shows the detailed annotation information of this database. Besides, when choosing records, if there were serious signal quality problems within the first 5-minute episode, the following 5-minute episode was used and a 0.05–45 Hz bandpass filter was applied for denoising for each record we chose before importing into the algorithm. We do not implement RR interval adjustment because *R* peaks were detected by *jqrs* method [21].

**Figure 1:**
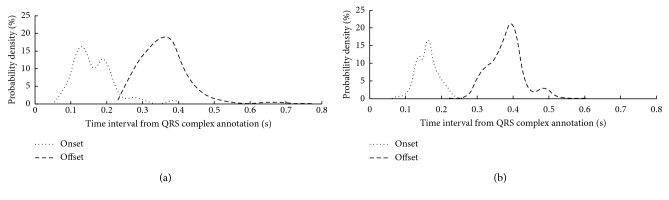
Probability density distribution of the time interval information between *T* wave onset/offset and *R* wave peak by analyzing the annotations from the two databases: (a) QT database and (b) European ST-T database.

To verify the consistency of the annotations between the two databases, we analyzed the time interval information between the *T* wave onset/offset and the corresponding *R* peak position for the two databases.  Figure 1 shows the probability density distributions of the time interval information from the two databases. As shown in Figure 1, we found that our manual annotations of the onset/offset of *T* wave in the European ST-T database had similar probability density distributions with the annotations in the QT database, which indicated the effectiveness of our annotations.

### 2.2. Sliding Window Area (SWA) Method

#### 2.2.1. SWA Method

**Figure 2:**
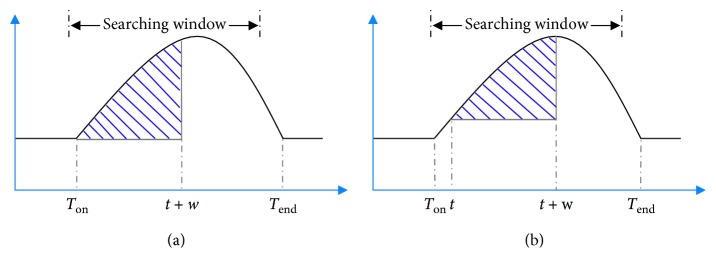
Demonstration of the SWA method for *T* wave onset detection.

**Figure 3:**
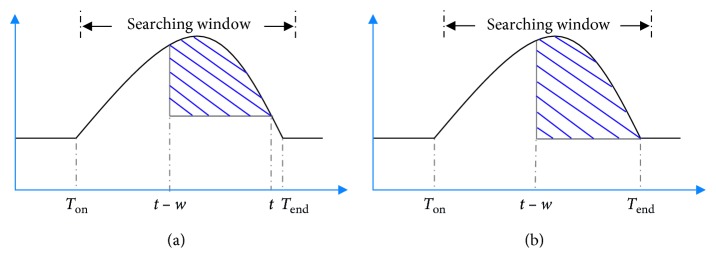
Demonstration of the SWA method for detecting offsets of the *T* wave.

SWA is an algorithm for detecting *T* wave onset and offset (*T*_on_ and *T*_end_, respectively) by analyzing the waveform area of ECG within a sliding window [31]. Onset/offset is detected when the area of the sliding window reaches its maximum in a prefixed searching range. Then, we show an example for explaining the method in Figures 2 and 3.

Figure 2 illustrates the detection for *T*_on_. Firstly, with the location of *R* peak, the left and right boundaries (*t*_1_ and *t*_2_, respectively) of search window are determined based on the current RR interval as suggested in the study by Song et al. [32]:

(1) $$\begin{array}{c} \left\{\begin{array}{cc} t_{1}=\left(\left\lceil 0.5\times \sqrt{{\text{RR}}_{i}}\right\rceil +R_{i}+0.08\right)\text{s}, & \\ t_{2}=\left(\left\lfloor 0.15\times \left({\text{RR}}_{i}\right)\right\rfloor +R_{i}+0.12\right)\text{s}, & \text{if }{\text{RR}}_{i}<0.88\text{ s},\\ t_{1}=\left(\left\lceil 0.5\times \sqrt{{\text{RR}}_{i}}\right\rceil +R_{i}+0.1\right)\text{s}, & \\ t_{2}=\left(R_{i}+0.32\right)\text{s}, & \text{if }{\text{RR}}_{i}\geq 0.88\text{ s}, \end{array}\right. \end{array}$$

where RR_*i*_ is the *i*_th_ RR interval and *R*_*i*_ is the *i*_th_ position of *R* peak.

The waveform area (area of onset denoted as: Ao) within the fixed sliding window $\left[\begin{array}{cc} t & t+w \end{array}\right]$ was calculated using the following formula:

(2) $$\begin{array}{c} \text{Ao}=\overset{t+w}{\underset{j=t}{\sum }}\left(s_{j}-{\bar{s}}_{k}\right), \end{array}$$

where *w*=0.12 s (by default), which is the window width, *t* stretches from *t*_1_ to *t*_2_, *s*_*j*_ is the waveform amplitude at the *j*_th_ sample point, and ${\bar{s}}_{k}$ is the local average amplitude (using a smoothing window of *p*=0.016 s by default), which is defined according to the following equation:

(3) $$\begin{array}{c} {\bar{S}}_{k}=\frac{1}{2p+1}\overset{t+p}{\underset{j=t-p}{\sum }}S_{j}. \end{array}$$

As shown in Figure 2, when *t* = *T*_on_, Ao reaches its maximum value.

Figure 3 illustrates the *T*_end_ detection. At first, with the location of the *R* peak, the left and right boundaries (*t*_3_ and *t*_4_, respectively) of the search window are determined based on the current RR interval as suggested in a study by Zhang et al. [31]:

(4) $$\begin{array}{c} \left\{\begin{array}{cc} t_{3}=\left(\left\lfloor 0.15\times {\text{RR}}_{i}\right\rfloor +R_{i}+0.148\right)\text{s}, & \\ t_{4}=\left(\left\lceil 0.7\times {\text{RR}}_{i}\right\rceil +R_{i}-0.036\right)\text{s}, & \text{if }{\text{RR}}_{i}<0.88\text{ s},\\ t_{3}=\left(R_{i}+0.28\right)\text{s}, & \\ t_{4}=\left(\left\lceil 0.2\times {\text{RR}}_{i}\right\rceil +R_{i}+0.404\right)\text{s}, & \text{if }{\text{RR}}_{i}\geq 0.88\text{ s}. \end{array}\right. \end{array}$$

The waveform area (area of ends denoted as: Ae) within the fixed sliding window $\left[\begin{array}{cc} t-w & t \end{array}\right]$ was then calculated according to the following formula:

(5) $$\begin{array}{c} \text{Ae}=\overset{t}{\underset{j=t-w}{\sum }}\left(s_{j}-{\bar{s}}_{k}\right), \end{array}$$

where *w*=0.128 s (by default), *t* is from *t*_3_ to *t*_4_, and *s*_*j*_ and ${\bar{s}}_{k}$ have been defined in equation (2). As shown in Figure 3, when *t* = *T*_end_, Ae reaches its maximum value. As for the difference between Figures 2 and 3 is the direction to calculate the sliding area.

In addition, Algorithm 1 shows the description of the traditional SWA algorithm and more details to which the algorithm proof can refer [31].

#### 2.2.2. Improved SWA Method

One key issue with respect to the SWA method is to accurately determine the search boundaries, but the search boundaries are closely related to the RR interval. As shown in Figures 2 and 3, if the interval of the searching window's boundaries was set too small which means that two boundary points are near the current *R* peak, the maximum of sliding area could not be found or the detected onset/offset of *T* wave are nearer to the *R* peak. These issues affect detection accuracy, which results in detection error and vice versa.

**Figure 4:**
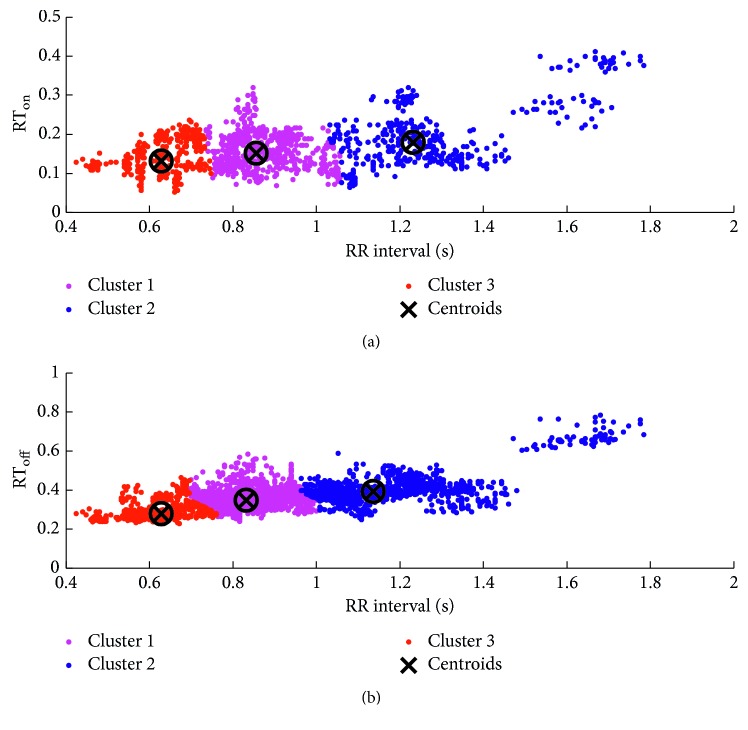
Clustering results for *T* wave feature points: (a) clustering information of *T* wave onsets; (b) clustering information of *T* wave offsets.

In the traditional SWA method, there are two piecewise functions with predefined parameter settings. In order to more accurately model the relationships between RR interval and the searching boundaries in this study, we performed a *k*-means clustering analysis between RR intervals and RT_on_ (RT_on_ denotes the time interval between the *R* peak and *T* wave onset) as well as the relationship between the RR intervals and RT_off_ (RT_off_ the time interval between the *R* peak and *T* wave offset), which is implemented by means of the *k*-means function in Matlab. The scatter plots with the optimal *k*-means clustering (*k* = 3) are shown in Figure 4 [38], and *k* is determined by combining the results of clustering and the computational complexity of parameters' settings as well as the adaptiveness of the algorithm. Then, the two relationships (between RR intervals and RT_on_, and between RR intervals and RT_off_) are obtained using the following equations:

(6) $$\begin{array}{c} \begin{array}{cc} \text{case }1\text{ : RR}<0.76\text{ s}, & 0.05\text{ s}<{\text{RT}}_{\text{on}}<0.25\text{ s},\\ \text{case }2\text{ : }0.76\text{ s}\leq \text{RR}<1.13\text{ s}, & 0.05\text{ s}<{\text{RT}}_{\text{on}}<0.35\text{ s},\\ \text{case }3\text{ : RR}\geq 1.13\text{ s}, & 0.05\text{ s}<{\text{RT}}_{\text{on}}<0.45\text{ s},\\ \text{case }1\text{ : RR}<0.72\text{ s}, & 0.2\text{ s}<{\text{RT}}_{\text{off}}<0.45\text{ s},\\ \text{case }2\text{ : }0.72\text{ s}\leq \text{RR}<1.1\text{ s}, & 0.2\text{ s}<{\text{RT}}_{\text{off}}<0.6\text{ s},\\ \text{case }3\text{ : RR}\geq 1.1\text{ s}, & 0.2\text{ s}<{\text{RT}}_{\text{off}}<0.8\text{ s}. \end{array} \end{array}$$

Thus, the three piecewise functions for determining the search boundaries for *T* wave onset and offset detections were obtained with the parameters presented in Table 2:

(7) $$\begin{array}{c} \left\{\begin{array}{cc} t_{1}=\left.\left(R_{i}+\left\lceil \text{ald}\times \sqrt{{\text{RR}}_{i}}\right\rceil +0.02\right)\right)s, & \\ t_{2}=\left(R_{i}+\left\lceil \text{alu}\times \sqrt{{\text{RR}}_{i}}\right\rceil +0.16\right)\text{s}, & \text{if }{\text{RR}}_{i}<0.76\text{ s},\\ t_{1}=\left(R_{i}+\left\lfloor \text{ard}\times \sqrt{{\text{RR}}_{i}}\right\rfloor +0.04\right)\text{s}, & \\ t_{2}=\left(R_{i}+\left\lfloor \text{aru}\times \sqrt{{\text{RR}}_{i}}\right\rfloor +0.24\right)\text{s}, & \text{if }0.76\text{ s}\leq {\text{RR}}_{i}<1.13\text{ s},\\ t_{1}=\left(R_{i}+\left\lceil \text{amd}\times \sqrt{{\text{RR}}_{i}}\right\rceil +0.04\right)\text{s}, & \\ t_{2}=\left(R_{i}+\left\lceil \text{amu}\times \sqrt{{\text{RR}}_{i}}\right\rceil +0.4\right)\text{s}, & \text{if }{\text{RR}}_{i}\geq 1.13\text{ s}, \end{array}\right. \end{array}$$

(8) $$\begin{array}{c} \left\{\begin{array}{cc} t_{3}=\left(R_{i}+\left\lceil \text{ald}\times {\text{RR}}_{i}\right\rceil +0.18\right)\text{s}, & \\ t_{4}=\left(R_{i}+\left\lceil \text{alu}\times {\text{RR}}_{i}\right\rceil +0.3\right)\text{s}, & \text{if }{\text{RR}}_{i}<0.72\text{ s},\\ t_{3}=\left(R_{i}+\left\lfloor \text{ard}\times {\text{RR}}_{i}\right\rfloor +0.18\right)\text{s}, & \\ t_{4}=\left(R_{i}+\left\lfloor \text{aru}\times {\text{RR}}_{i}\right\rfloor +0.4\right)\text{s}, & \text{if }0.72\text{ s}\leq {\text{RR}}_{i}<1.1\text{ s},\\ t_{3}=\left(R_{i}+\left\lceil \text{amd}\times {\text{RR}}_{i}\right\rceil +0.18\right)\text{s}, & \\ t_{4}=\left(R_{i}+\left\lceil \text{amu}\times {\text{RR}}_{i}\right\rceil +0.48\right),\text{s} & \text{if }{\text{RR}}_{i}\geq 1.1\text{ s}. \end{array}\right. \end{array}$$

**Figure 5:**
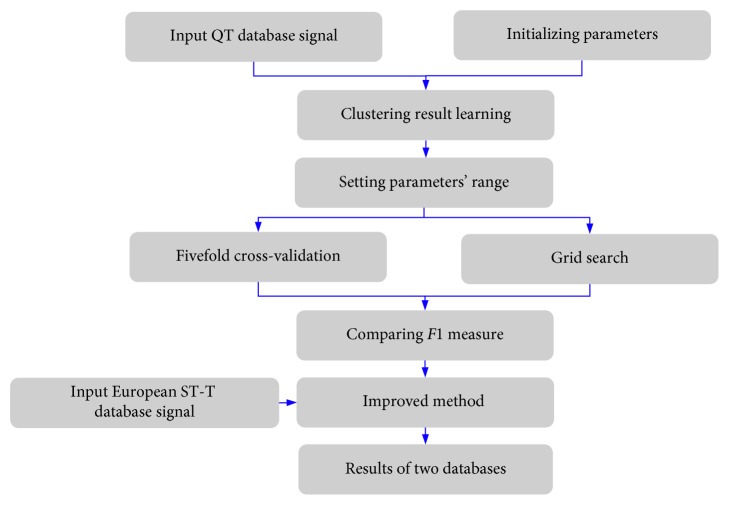
The block diagram of the proposed method for delineating the *T* wave onset/offset.

Then, the *grid search* was used to determine the best combination of parameters in equations (7) and (8), which was implemented by for loop. In a loop, we changed the value of one parameter at a time, kept the other parameters unchanged, and applied the algorithm in the QT database as well as using a 5-fold cross-validation. Then, we stored the *F*1 measure of one loop and started another loop. Through all loops, we traversed all of the combinations of parameters referred to in Table 2. After comparing the results, the combinations of parameters with the highest *F*1 measure were chosen. The best parameters' combinations for *T* wave onsets are listed: ald = 0.4, alu = 0.2, ard = 0.4, aru = 0.4, amd = 0.3, and amu = 0.0 and for *T* wave ends are listed: ald = 0.2, alu = 0.1, ard = 0.2, ard = 0.1, aru = 0.0, amd = 0.0, and amu = 0.1. The improved SWA method can be summarized as a block diagram in Figure 5.

### 2.3. Evaluation Method

Detections for true and false positives (TP and FP, respectively) and false negative (FN) were determined with a threshold of 100 ms. In this study, indices like sensitivity (Se), positive precision (*P*+), and *F*1 measurement were selected as evaluation indices [39, 40] with the following definitions: Se=TP/(TP+FN), *P*+=TP/(TP+FN), and *F*1=(TP × 2)/(TP × 2+FN+FP). *F*1 measure is selected other than accuracy since *F*1 measure is the weighted average of precision and recall which satisfies our asymmetric datasets where values of false positive and false negatives are not the same.

## 3. Results

**Figure 6:**
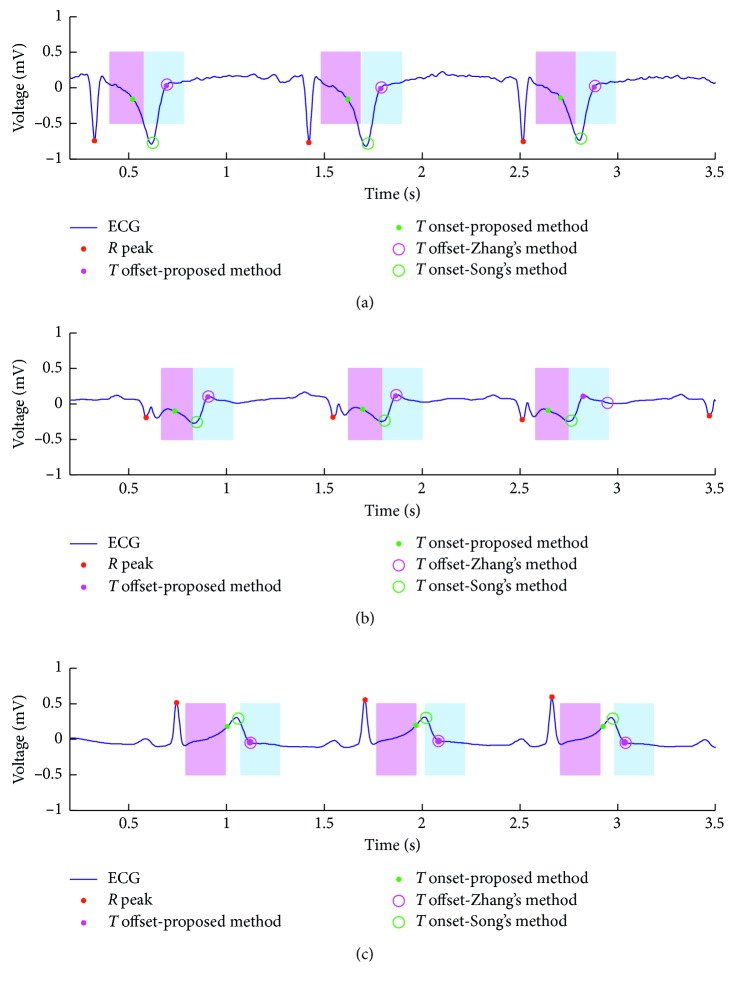
*T* wave detection examples. The solid points marked (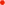) are *R* peaks; the solid points marked (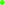 and 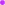) are results of our method; the hollow circles marked (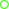 and 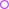) are results of the traditional method; the shadow areas are accepted as TP cases. (a) e0107; (b) e0111; (c) e0118.

Figure 6 shows the detection examples of the proposed method, compared with the traditional methods, Zhang's method for *T* wave offset detection [31] and Song's method for *T* wave onset detection [32].  Figure 6(a) shows the inverted *T* wave detections, Figure 6(b) shows the biphasic *T* wave detections, and Figure 6(c) shows the normal *T* wave detections. From Figure 6, *T* wave offset detections get better results than *T* wave onset detections. And, our method got obviously better results when it is applied in *T* wave onsets detections.

### 3.1. Results from the QT Database

We firstly tested the performance of the improved SWA method on the QT database. The traditional SWA methods (Song's method [32] and Zhang's method [31]) were used as comparators.

Table 3 shows the results of onset and offset detections in the QT database. Both of the two channels signals (first and second channels) were tested. From Table 3, we found the improved SWA method significantly enhanced detection accuracies for both onset and offset detections. For onset detection, *F*1 improved from 54.70% to 70.46% and 54.05% to 72.94% for two ECG channels, respectively. For offset detection, *F*1 improved from 87.83% to 93.73% and 86.73% to 94.75% for two ECG channels, respectively. In addition, detection errors were also analyzed. As expected, the improved SWA method indicated smaller detection errors than the traditional method except for a slight increase in the offset detection from the second channel (traditional 0.027 ± 31.85 ms versus improved 2.45 ± 33.98 ms). However, it is worthwhile to note that all Se, *P*+, and *F*1 indices increased from ∼86% to ∼94%.

### 3.2. Results from the European ST-T Database

Table 4 shows the results of onset and offset detections in the European ST-T database. The improvements after using the improved method were more significant when performing *T* wave onset detection. *F*1 improved from 41.02% to 84.13% and 44.33% to 87.62% for two ECG channels, respectively. The mean detection errors significantly decreased from 19.52 ms to 7.04 ms and 36.27 ms to 6.35 ms for two ECG channels, respectively. Performance improvements in offset detection were small but convincing *F*1 improved from 98.83% to 99.57% and 91.76% to 98.29% for two ECG channels, respectively. However, the mean detection errors for *T* wave offset detection slightly increased (not significant) when performing the improved method.

## 4. Discussion

As seen from Tables 3 and 4, both *T* wave onset and offset detection of the new proposed method reported better performances (*F*1 measure) than the traditional method, suggesting that applying the clustering technique in the SWA method for deciding searching boundaries is helpful to enhance detection accuracy. In addition, clustering is a statistical-based technique, which can be used to determine whether the independent part of a population belongs to different groups by comparing quantitative multiple features [38]. Besides, we noted that, for the *T* wave offset detections, neither the traditional SWA nor the improved version reported the better performance than the detection of *T* wave onsets. One possible explanation is that Zhang proposed this method originally to detect *T* wave offsets not *T* wave onsets and proved its mathematical rationality for *T* wave offsets. Another possible explanation is that the clustering method for determining the search boundaries is a statistical-based technique. Therefore, the accuracy of the clustering results is related to the data amount. However, the annotated *T* wave onsets in the QT database are far less than the annotated *T* wave offsets (1371 versus 3452). Thus, the relationship found by clustering analysis between the RR interval and RT_on_ is not that strong (Figure 4(a)) compared to the relationship between the RR interval and RT_off_ (Figure 4(b)). Moreover, the significant difference between the improved and traditional SWAs indicates that the improved version can more extensively and adaptively determine the search window's boundaries by using the *k*-means clustering based on the QT database and grid search strategy. However, the traditional SWA only used predefined parameters and did not give out any detailed explanations.

Another difference between Zhang's [31] and our results was observed when using the QT database for validation, Zhang's study chose the better result from the outputs of the two ECG channels [31]. In order to compare our results with those from Zhang, we also calculated smaller errors from the results of two ECG channels. The comparable results are summarized in Table 5. The mean detection errors are similar between Zhang's and our results. We also noted that the standard deviation of detection errors was 25.82 ms for our method and 21.19 ms for the traditional SWA. Both of them were smaller than the acceptable threshold (30.6 ms) proposed by the common standards in Electrocardiography Working Party [41].

Table 5 also summarizes comparable results from other studies. The wavelet-based method reported a mean error of 1.6 ms [13, 14]; the low-pass differentiation-based method gave a relative large mean error of 13.5 ms [20], while the hidden Markov model-based method reported a mean error of 5 ms [21, 22]. Furthermore, the partially collapsed Gibbs sample reported 4.3 ms [23], and the *k*-nearest neighbor-based method reported 2.8 ms [20]. The TU complex analysis gave a minimum detection mean error of 0.8 ms but did not include the corresponding Se and *P*+ results [28]. In addition, methods proposed by Mahsa with linear and nonlinear phase observation to detect fiducial points are also listed as comparative method [42], and two parts of QT database including normal sinus rhythm and arrhythmia database are used when evaluating extraction of fiducial points and the nonlinear observation has more smaller deviations 23 ms for the first database and 19 ms for the second database.

The potential issues existing in the above studies consist of two main points: (1) the time tolerance for determining true positive detection was not clear and (2) training and testing were both performed in the QT database, but we used the European ST-T database as the independent testing dataset.

Besides, as deep-learning technology improves, more and more methods based on this technique have been proposed to detect ECG feature points; for instance, a recently proposed method using neural network and fixed-size least-squares SVM to detect *T* wave end reported it is a minimum detection mean error of −3 ms in the QT database; a QRS complex detection by using two-level convolutional neural network [8] reported its sensitivity of 99.77% in the MIT-BIH AR database. When using deep-learning technique, a great amount of data is needed, and in *T* wave detection, the annotated *T* wave ends are limited but a meaningful strategy was proposed in [19], which is to use different strategies for selecting different training sets such as random selection and *k*-means. But, we just proposed an idea that is to use one independent database (QT dataset) as the training set and testing set and another independent database (records from the European ST-T database annotated by a trained staff) as the testing set.

**Figure 7:**
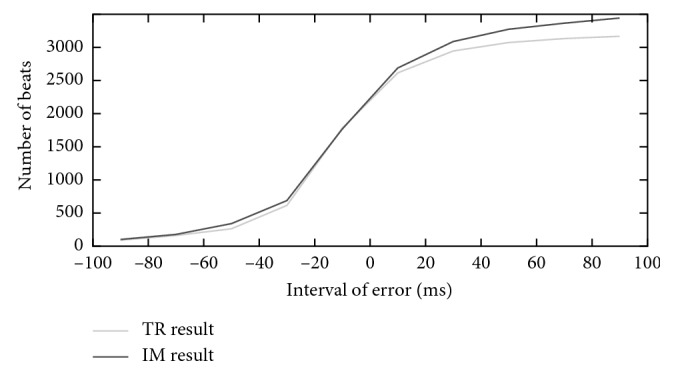
The cumulative line chart of error (*T* offsets in the QT database). TR result represents the traditional SWA method result, and IM result represents the improved SWA method result.

In order to illustrate difference between error and *F*1 measure, we did statistical analysis of error. And, Figure 7 gives the cumulative line chart of error (denoted as CLCE) of *T* wave offsets in the QT database which explains our method got more true positive beats than the traditional method inside our time tolerance. The CLCE of *T* wave onsets in the QT database and CLCE of *T* wave in the European ST-T database also have the same regularities of distribution as it did in the *T* wave offsets in the QT database.

Moreover, the limitation of our study is that the annotations of the European ST-T database are only done by a trained staff member which may result in nonauthoritative annotations. Besides, we only combine the data statistic and data mining technique to changing the parameters of traditional SWA method. In our following work, more records with authoritative annotations will be used to test the robustness of the combination of parameters we obtained in this study.

## 5. Conclusion

In this paper, an improved sliding window area method for detecting *T* wave onset and offset was proposed. The main contribution/novelty was for application of the data statistic and data mining technique: (1) *k-means clustering* for the setting of search boundaries and (2) *grid search strategy* to optimize the parameters. Experiments performed in the QT database and the European ST-T database demonstrated the improved method's better performance.

## Figures and Tables

**Algorithm 1:**
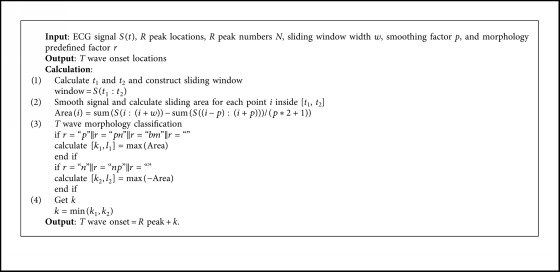
Traditional SWA algorithm (*T* wave onset detection).

**Table 1:** Summary of the annotative information of the QT and the European ST-T databases.

Variable	QT database		European ST-T database	
	Onset	Offset	Onset	Offset
No. of recordings	43	105	23	23
No. of annotated beats	1371	3542	14337	14337
Min. Dis_qrs (ms)	52	228	60	220
Max. Dis_qrs (ms)	412	784	264	612
Mean of Dis_qrs (ms)	164	360	160	380
SD of Dis_qrs (ms)	60	71	29	47

Dis_qrs: the time interval between the *T* wave onset/offset and the *R* wave position within the current beat; SD: standard deviation. The annotations of the QT database are taken from the database website https://www.physionet.org/physiobank/database/qtdb/doc/index.shtml.

**Table 2:** Information of parameters when detecting the *T* wave.

Parameters	*T* onset			*T* offset		
	IV	CS	CR	IV	CS	CR
ald/alu/ard	0.1	0.1	0.1∼0.4	0.1	0.1	0.1∼0.4
aru/amd				0.0		0.0∼0.4
amu	0.0		0.0∼0.4			

IV: initialized value; CS: change step; CR: change range.

**Table 3:** Results of *T* wave detection in the QT database.

Detection	Channel	Method	Se (%)	*P*+ (%)	*F*1 (%)	Error mean ± SD (ms)
Onset	First	Traditional SWA [32]	54.70	54.70	54.70	−30.2 ± 40.75
		Improved SWA	70.46	70.46	**70.46**	**7.3** **±** **53.12**
	Second	Traditional SWA [32]	54.05	54.05	54.05	−36.27 ± 43.29
		Improved SWA	72.94	72.94	**72.94**	**6.35** **±** **53.78**

Offset	First	Traditional SWA [31]	87.83	87.83	87.83	−2.57 ± 30.08
		Improved SWA	93.93	93.93	**93.93**	**1.19** **±** **33.59**
	Second	Traditional SWA [31]	86.73	86.73	86.73	0.027 ± 31.85
		Improved SWA	94.75	94.75	**94.75**	**2.45** **±** **33.98**

**Table 4:** Results of *T* wave detection in the European ST-T database.

Detection	Channel	Method	Se (%)	*P*+ (%)	*F*1 (%)	Error mean ± SD (ms)
Onset	First	Traditional SWA [32]	41.02	41.02	41.02	19.52 ± 31.89
		Improved SWA	84.13	84.13	**84.13**	**−7.87** **±** **44.22**
	Second	Traditional SWA [32]	44.33	44.32	44.33	−36.27 ± 43.29
		Improved SWA	87.62	87.61	**87.62**	**−15.20** **±** **33.54**

Offset	First	Traditional SWA [31]	98.80	98.86	98.83	22.2 ± 22.70
		Improved SWA	99.50	99.65	**99.57**	**26.94** **±** **20.98**
	Second	Traditional SWA [31]	91.80	91.71	91.76	21.07 ± 26.31
		Improved SWA	98.29	98.28	**98.29**	**24.54** **±** **25.52**

**Table 5:** Comparable detection results of *T* wave offset in the QT database.

Methods	Annotations	Se (%)	*P*+ (%)	Mean ± SD (ms)
Improved SWA	3542	**98.5**	**98.5**	**1.21** **±** **25.82**
Traditional SWA [31]	3542	95.5	95.5	−1.12 ± 21.19
Wavelet-based [13, 14]	3542	99.77	97.79	−1.6 ± 18.1
Low-pass differentiation-based [20]	3542	99.00	97.74	13.5 ± 27.0
Hidden Markov model-based [21, 22]	3542	NA	NA	−5 ± 14
Partially collapsed Gibbs sample [23]	3403	99.81	98.97	4.3 ± 20.8
*k*-nearest neighbor-based [30]	30 records	NA	NA	2.8 ± 18.6
TU complex analysis [28]	3528	92.60	NA	0.8 ± 30.3
Neural network and fixed-size least-squares SVM [19]	3542	NA	NA	−3.0 ± 16.9
L.EKF25 [42]	10 records	NA	NA	11 ± 39
N.L.EKF25 [42]				4 ± 23
L.EKF25 [42]	15 records	NA	NA	−17 ± 30
N.L.EKF25 [42]				−21 ± 19

NA: not available; L.EKF25: linear Kalman filter; N.L.EKF25: nonlinear Kalman filter.

## Data Availability

The data used to support the findings of this study are available from the corresponding author upon request.
